# Orbiting, colliding, and merging liquid lenses on a soap film: Toward gravitational analogs

**DOI:** 10.1093/pnasnexus/pgag079

**Published:** 2026-03-24

**Authors:** Jean-Paul Martischang, Benjamin Reichert, Ilies Haouche, Germain Rousseaux, Alexis Duchesne, Michael Baudoin

**Affiliations:** Univ. Lille, CNRS, Centrale Lille, Univ. Polytechnique Hauts-de-France, UMR 8520, IEMN, Lille F59000, France; Univ. Lille, CNRS, Centrale Lille, Univ. Polytechnique Hauts-de-France, UMR 8520, IEMN, Lille F59000, France; Univ. Lille, CNRS, Centrale Lille, Univ. Polytechnique Hauts-de-France, UMR 8520, IEMN, Lille F59000, France; Institut Pprime (UPR 3346), CNRS - Université de Poitiers - ISAE ENSMA, 11 Boulevard Marie et Pierre Curie Téléport 2 - BP 30179, Futuroscope Chasseneuil Cedex 86962, France; Univ. Lille, CNRS, Centrale Lille, Univ. Polytechnique Hauts-de-France, UMR 8520, IEMN, Lille F59000, France; Univ. Lille, CNRS, Centrale Lille, Univ. Polytechnique Hauts-de-France, UMR 8520, IEMN, Lille F59000, France; Institut Universitaire de France, 1 rue Descartes, Paris 75005 France

**Keywords:** soap film, gravito-capillary interactions, analog gravity

## Abstract

Gravity governs the large-scale structure of the Universe, driving the formation and interactions of galaxies. These interactions generate distinctive features—such as complex orbits, tidal spiral arms, and bridges—that are commonplace at astrophysical scales but rarely observed at human scales. Multibody dynamics can be observed at laboratory scale with particles at liquid interfaces interacting via the “Cheerios effect,” but such systems have limited ability to reproduce gravity-shaped structures because of their short-range interactions, strong dissipation, and a limited number of rigid bodies. Here, we show that miscible millimetric water lenses on a soap film can sustain long-lived orbital motion, collisions, and mergers, producing tidal arms and bridges reminiscent of interacting galaxies. These dynamics arise from a Newton-like gravito-capillary attraction, low dissipation, and lens deformability. A quantitative model of film deformation accurately predicts both static lens shapes and orbital trajectories for single and multiple bodies. This controllable, time-resolved platform enables direct experimental study of the gravity-driven formation of complex, deformable structures, paving the way for laboratory gravitational analogs.

Significance statement Gravity shapes the Universe, from the motion of planets to the interactions of galaxies. But, these processes unfold over millions to billions of years—far beyond human observation. We have developed a tabletop experiment where millimetric, deformable liquid lenses orbit, collide, and merge on a soap film under a 2D Newton-like gravito-capillary attraction. Long-range attractive forces, lens deformability, and minimal energy loss allow them to sustain long-standing orbits and form tidal arms and bridges, resembling their galactic counterparts. Our mathematical model quantitatively predicts their shapes and multibody orbital dynamics. This simple system offers a way to study gravity-driven formation of complex structures in real time, drawing analogies between phenomena occurring at vastly different length and time scales.

## Introduction

Our universe is shaped by gravitational interactions. Yet the observation of the dynamical organization of the matter at the astrophysical scales lies beyond the reach of human time-scale observation. This lack of chronological data contributes to some persisting mysteries surrounding the formation and interaction of galaxies (1–4). At the laboratory scale, multiple-body gravity-driven interactions have been widely studied in the field of interfacial fluid dynamics. Indeed, millimetric identical particles deposited over a liquid/air interface experience gravito-capillary induced interparticle attractive forces resulting from the distortion of the interface (5–7). This effect, commonly referred as the “Cheerios effect,” is at the core of capillary self-assembly of micro-objects (8), used in a wide range of applications ranging from the assembly of microcomponents to the synthesis of functional materials and additive manufacturing (9). Recently, capillary orbits resembling celestial systems have also been reported (10, 11). Yet, these simple systems based on a limited number of nondeformable particles cannot give rise to complex structures, such as the ones resulting from the interactions of galaxies.

In this article, we investigate the orbit, collision, and merging of miscible liquid droplets supported by a soap film, hereafter referred to as “lenses”. We first show that depositing a liquid drop at the center of a soap film produces a stable, lens-shaped structure with a universal radius. This shape results from the balance between gravitational forces and capillary forces induced by the film’s deformation. Second, we examine the dynamics of a single lens on the fluid membrane, and reveal that the latter acts as a harmonic potential gravito-capillary trap for the suspended lens. Third, we consider the two-body (lenses) problem on the soap film. The fluidic nature of the system lowers significantly the drag experienced by the lenses, which exhibit sustained orbital motion governed by the background harmonic well on the one hand, and a gravito-capillary pair attraction on the other hand. It appears that the long ranged nature of the latter effect coupled to lens deformability allows to evidence physical effects usually observed within the astrophysical realm such as tidal force related deformation and complex merging patterns characteristic of galaxies.

This work paves the way toward in-lab study of the dynamics of the formation of gravity driven complex structures akin to those observed at the astrophysical scale, hence bringing an original contribution to the growing field of Analogue Gravity (12–19).

## Methods

**Figure 1:**
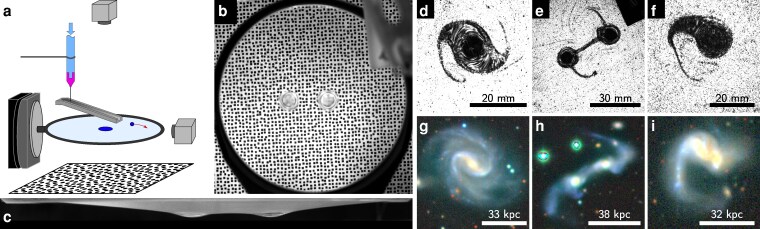
Liquid lenses orbiting and merging on a soap film and resulting structures. a) Sketch of the experimental setup. b and c) Unprocessed images of two orbiting lenses on a soap film from above (see also Movie S1) and from aside, respectively. d–f) Images of structures resulting from the interactions of two orbiting lenses and comparison to similar galaxy structures (g–i). d) and e) feature the interaction of lenses of identical mass (22.5 and 18.4 mg, respectively), whereas f) illustrates the interaction of lenses of different masses (38.5 and 15.5 mg, respectively). Images g–i) correspond to the three galaxies ARP 73, ARP 238, and ARP 55, respectively, taken from the Legacy Surveys / D. Lang (Perimeter Institute) (20).

In the present work, a soap film is formed by immersing a 3D-printed, 10-cm circular frame into an aqueous surfactant solution (20${\%}_{v}$—volumetric percent—glycerol and SDS at 5 g·L^−1^) and then pulling it out. The frame, whose shape has been designed to avoid spurious fluid accumulation on the periphery, is maintained horizontal with a goniometer fixed on an anti-vibration table and kept in a sealed box at 80 to 90% relative humidity to increase the soap film lifetime. Then, droplets of de-ionized water are injected with a syringe either directly on the soap film or using a hydrophobic slide to impart a controlled initial horizontal lens velocity (Fig. 1a). The mass of the lens is adjusted by changing the diameter of the nozzle. Each of the 18 available diameters was thoroughly tested by depositing around 50 lenses on a precision balance, revealing standard deviations in the masses of 2 to 16% of the observed means, with a median at 5% of the mean masses. The soap film “in-plane” trajectories of the lenses (Fig. 1b) and their profile and vertical movements (Fig. 1c) are recorded with two orthogonal cameras. Images from the upper camera were processed either by using the schlieren software ComBOS (21) (experimental images in color), or by applying a sliding Laplacien and then a standard deviation filter on them (all experimental images unless explicitly specified). As the focus was made on a highly detailed background speckle behind the soap film, this method allowed to highlight well defined blurry regions, hence the places were the interfaces of the film varied strongly, revealing the positions and shapes of the lenses.

## Results

### Profile of a single, centered liquid lens

**Figure 2:**
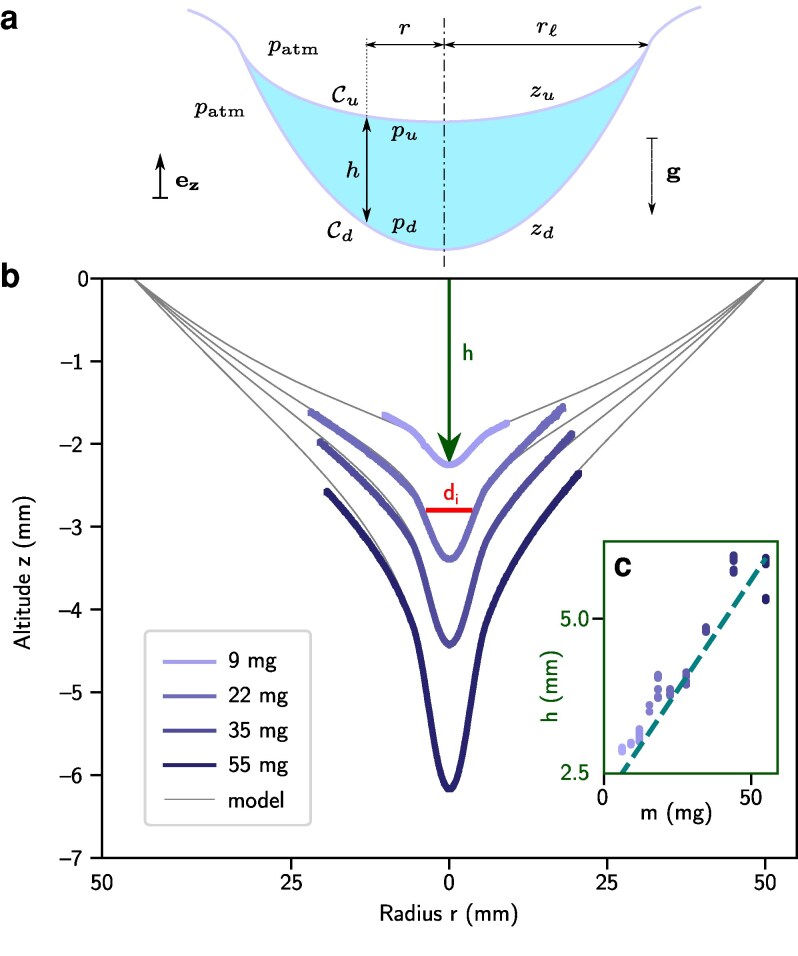
Profiles of a soap film deformed by a water droplet (lens) deposited at its center. a) Sketch of the lens and introduction of the parameters of the problem. b) Superimposed profiles of the bottom interface of the lens captured with a camera for different lens masses (thick purple lines) and comparison with theoretical prediction obtained from Eqs. 2 and 5 (thin gray lines). The thick red line with the notation $d_{i}$ at the top indicates the distance between two inflexion points of a given profile. Inset: Evolution of the lowest altitude *h* of the lens (points) as a function of the droplet mass and comparison to theory (dashed line), corresponding to $h=|z_{d}(0)|$ in Eq. 5.

When the droplet is deposited at the center of the soap film with a syringe, the liquid remains confined in a central thick region with a “lens” shape, whose weight induces a global deformation of the surrounding thin soap film acting as a fluidic “membrane” (Fig. 2a). This profile evolves slowly, on characteristic time scales (22) much longer than the present experiments and will hence be considered in the following as quasistatic. The persistence and shape of this lens as well as the global deformation of the soap film membrane can be rationalized by considering the equilibrium between gravity and capillary forces at play:

(i) The equilibrium profile of the fluidic membrane in the peripheral region $r\in [r_{\ell },R]$ can be obtained from a force balance on the soap film, considering the capillary force, the weight of the membrane, and the lens load as a point central force of intensity *mg* (see Supplementary material for details):


(1) 2σΔz(r)=ρgξ+mgδ(r),

with *r* the radial distance from the center of the soap film, $r_{\ell }$ the radius of the lens, *R* the radius of the frame, *z* the vertical displacement of the membrane, *ξ* the thickness of the soap film considered here as constant, *σ* the surface tension, *m* the injected droplet mass, *ρ* the liquid density, *g* the gravitational acceleration, and *δ* the Dirac delta function. The resulting profile obtained from the resolution of this linear equation is a superposition of a catenoid induced by the point load, and a parabola emerging from the membrane weight:


(2) $$z(r)=\frac{m\;g}{4\;\pi \;\sigma }\ln\;\left(\frac{r}{R}\right)+\frac{\xi }{8\;\ell_{c}^{2}}\;(r^{2}-R^{2}).$$

(ii) The upper and bottom interface profiles in the central thick region ($r\in [0,r_{\ell }]$), where the lens lies, can be derived from a vertical pressure balance considering Laplace and hydrostatic pressures across the lens:


(3) $$\sigma \left[C_{u}(r)+C_{d}(r)\right]=\rho g\;\left[z_{u}(r)-z_{d}(r)\right],$$

with $C_{u}(r)$ and $C_{d}(r)$ the upper and lower curvatures of the lens at distance *r* from the center of the film and $z_{u}$ and $z_{d}$ the upper and lower altitudes of the lens, see Fig. 2a. The resolution of this equation with a matching of the altitude and slope of the inner and outer solutions for $r=r_{\ell }$ leads to the following expressions of the upper and lower interfaces:


(4) $$z_{u}(r)=\frac{J_{0}(\eta )}{J_{1}(\eta )}\;\left(\frac{mg}{4\pi \sigma \eta }+\frac{h\eta }{4}\right)\;\left(1-\frac{I_{0}(r/\ell_{c})}{I_{0}(\eta )}\right)+z(r_{\ell })$$

(5) $$z_{d}(r)=\frac{J_{0}(\eta )}{J_{1}(\eta )}\;\left(\frac{mg}{4\pi \sigma \eta }+\frac{h\eta }{4}\right)\;\left(1-\frac{J_{0}(r/\ell_{c})}{J_{0}(\eta )}\right)+z(r_{\ell }),$$

with $\eta =r_{\ell }/\ell_{c}$, $J_{0},J_{1},I_{0}$ the Bessel functions of zeroth and first orders and $\ell_{c}=\sqrt{\sigma /\rho \;g}$ the capillary length. These results are obtained within the linearized approximation of the curvature.

The composite solution of the bottom interface profile obtained from the combination of Eq. 5 for $r\in [0,r_{\ell }]$ and Eq. 2 for $r\in [r_{\ell },R]$ shows excellent quantitative agreement with the one measured experimentally from side view imaging in Fig. 2b for different droplet masses, with no fitting parameters aside from a unique vertical offset—the same for all points—hence confirming that the physics is well captured by this simple model. The following values for the surface tension and film thickness were considered, measured respectively with the pendant drop method and reflectance spectrophotometry (23): $\sigma =\;34\;\text{mN}\cdot {\text{m}}^{-1}$ and 15μm, the latter corresponding to an average value of the measured thickness spanning between 10 and 20μm. The experimental maximum depth $h=|z_{d}(0)|$ is plotted in insert of Fig. 2b as a function of the droplet mass, alongside a theoretical prediction (dashed line) obtained by evaluating $\left|z_{d}(r=0)\right|$ in Eq. 5. The predicted linear dependency shows good agreement with the experimental data.

In these equations, the radius $r_{\ell }$ is determined through a mass balance $m=\rho \int_{r=0}^{r_{\ell }}2\pi [z_{u}(r)-z_{d}(r)]\;r\;dr$ leading to the implicit equation:


(6) $$\frac{J_{0}(r_{\ell }/\ell_{c})}{J_{1}(r_{\ell }/\ell_{c})}=-\frac{I_{0}(r_{\ell }/\ell_{c})}{I_{1}(r_{\ell }/\ell_{c})}.$$

A striking feature in this expression (also observed experimentally) is that the lens radial extension $r_{\ell }$ (≈6mm) does not depend on the mass of the lens.

Following the deposition of the drop on the soap film, gravity drainage starts to occur toward the central lens. This phenomenon creates surface tension gradients due to an uneven surfactant concentration at the interface (24, 25). In our theoretical analysis, this effect is not considered. This assumption is verified a posteriori since the experimental profile of the film is quantitatively recovered by our model in which the equilibrium value of surface tension is considered (Fig. 2b).

### Dynamics of a single off-centered lens

**Figure 3:**
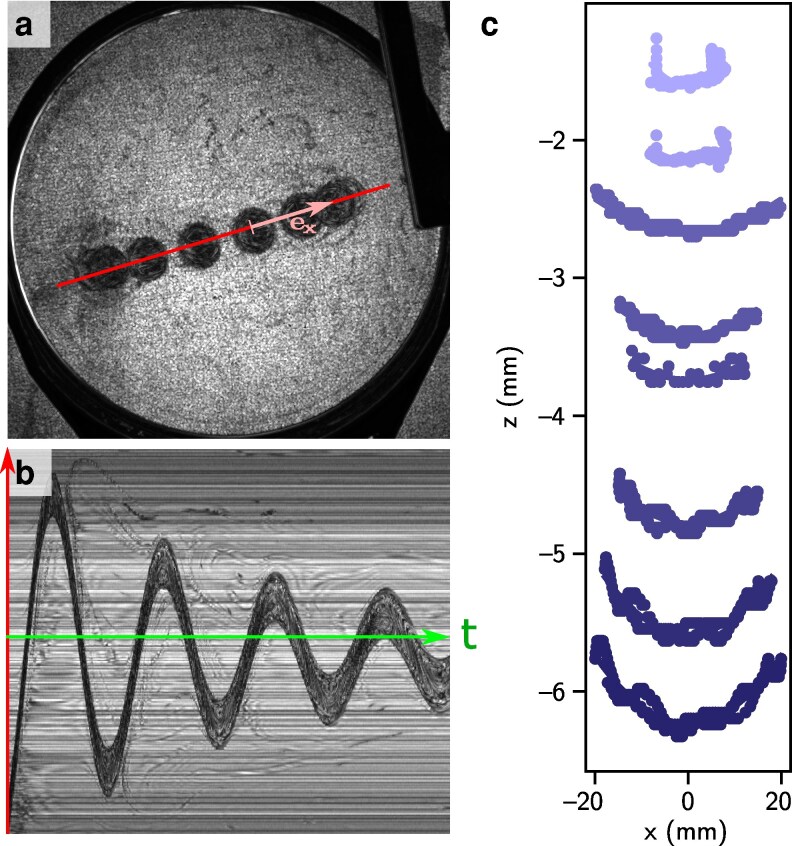
Single lens moving on a soap film. a) Superposition of different positions of the lens. The solid line shows the axis of the lens’s motion. b) Temporal evolution of the “in plane” position of the lens along this line. The lens motion follows the dynamics of a damped harmonic oscillator. c) Side view trajectories of lenses of increasing masses (clearer to darker) 6, 9, 28, 31, 35, 38, 51, and 55 mg.

Now, if the droplet is injected at an off-center position without initial horizontal velocity, the lens starts oscillating along an axis $e_{x}$ as shown on Fig. 3a and b, and Movie S2. Laterally, i.e. in the (Oxz) plane, the lens trajectory is parabolic (see Fig. 3c). The lens therefore oscillates in a harmonic energy well imposed by the bounded soap film. This trajectory can simply be obtained by generalizing Eq. 1 to an off-center position of the lens $r_{0}$, i.e. by replacing z(r) by $z(r,r_{0})$ and δ(r) by $\delta (r-r_{0})$, with r the radius vector and $r_{0}$ the position of the lens. Since the lens oscillates along the axis (Ox), its position is a function only of the coordinates $x_{0}$ and $z_{0}$. The relation between these two parameters defining the lens trajectory can be obtained by solving generalized Eq. 1 (see Supplementary material for detailed calculation):


(7) $$z_{0}(x_{0})=\frac{1}{8}\frac{\xi }{\ell_{c}^{2}}(x_{0}^{2}-R^{2})\;+\;\frac{m\;g}{4\pi \sigma }\;[\ln\;(r_{\ell }/R)+(x_{0}/R{)}^{2}].$$

**Figure 4:**
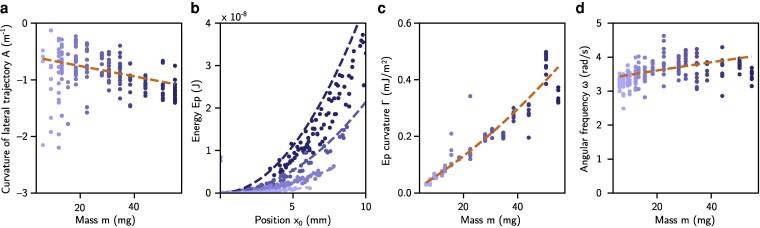
Parameters derived from a lens moving horizontally on a soap film. The experimental points are superimposed with the models developed in this work, in dashed lines. a) Curvature *A* of the vertical trajectory $z=Ar_{0}^{2}$ against the lens mass *m*. b) Total energy of the system according to the radial position of the lens for masses 6, 16, 31, and 55 mg (smaller masses are displayed in lighter shades). The *Γ* coefficient of Eq. 14 is measured from a quadratic fit of the experimental data, and displayed in (c). The expression of the coefficient *Γ* from Eq. 14 is indicated as a dashed line. d) Pulsation of the horizontal oscillation of the lens.

The agreement between this equation and the trajectories observed in Fig. 3c is illustrated in Fig. 4a where the experimental curvature of the lateral trajectory is plotted with respect to the lens mass, and compared to the trajectory curvature predicted from Eq. 7: $A=\frac{1}{8}\frac{\xi }{\ell_{c}^{2}}+\frac{m\;g}{4\pi \sigma R^{2}}$. Note that this predicted trajectory relies on two underlying assumptions: (i) the membrane is considered at equilibrium and hence the inertial force in the liquid membrane is neglected and (ii) the inertial force exerted on the lens in the vertical direction is also neglected.

Now that we have determined the lateral lens trajectory, we can focus on the harmonic oscillatory motion of the lens in the *x* direction. Projecting Newton’s law $m\;\ddot{r_{0}}=F_{\sigma }+F_{d}+m\;g$ onto the base vectors $e_{x}$ and $e_{z}$, respectively leads to:


(8) $$m\;\ddot{x_{0}}=F_{\sigma }\cdot e_{x}+F_{d}\cdot e_{x},$$

(9) $$0=F_{\sigma }\cdot e_{z}-mg,$$

with $F_{\sigma }$ the capillary restoring force and $F_{d}$ the drag force, which, in the viscous regime, will be proportional and opposite to the velocity vector. This expression is obtained in the low-slope approximation, hence neglecting the speed and acceleration in the *z* direction. If we now introduce the angle $\beta =∠(e_{z},F_{\sigma })$, we obtain straightforwardly $F_{\sigma }\cdot e_{x}=\tan\;(\beta )mg$. Hence we just need to determine the angle *β* to identify $F_{\sigma }$. This can be achieved (cf. Supplementary material) by considering the profile of the deformed membrane for an off-center lens, resulting in:


(10) $$\tan\;\beta =\frac{\xi x_{0}}{4\ell_{c}^{2}}+\frac{m\;g}{4\;R\;\pi \sigma }\left[\frac{x_{0}/R-x_{0}^{3}/R^{3}}{(1-x_{0}^{2}/R^{2}{)}^{2}-(x_{0}\;r_{\ell }/R^{2}{)}^{2}}\right],$$

which is valid in the limit of small lenses compared to the support radius ($r_{\ell }\ll R$). The expression for $F_{\sigma }$ then reduces to:


(11) $$F_{\sigma }\cdot e_{x}=-\Gamma \;x_{0},$$

with the supporting film effective spring stiffness


(12) $$\Gamma =\left[\frac{mg\xi }{4\ell_{c}^{2}}+\frac{(mg{)}^{2}}{4\pi \sigma R^{2}}\right].$$

If we introduce a damping factor $F_{d}\cdot e_{x}=-c\dot{x_{0}}$ related to the drag (not determined theoretically in the present work), we obtain from Eq. 8 the celebrated damped harmonic oscillator equation:


(13) $$\ddot{x_{0}}+2\alpha \dot{x_{0}}+\omega_{0}^{2}x_{0}=0,$$

with $\omega_{0}=\sqrt{\Gamma /m}$ the natural angular frequency and α=c/2m the damping coefficient. From this calculation, we can introduce a potential energy associated with the lens lateral movement on the soap film:


(14) $$E_{p}=\frac{1}{2}\;\Gamma x_{0}^{2}\;.$$

This model can now be compared to the experiments conducted using various lens masses. First, we take advantage of the damping mechanism to extract the evolution of the potential energy as a function of the radius $x_{0}$. Indeed, the extremal position reached by the lens $\left|x_{\max}^{k}\right|$ during its *k*^th^ half-turn on the film decreases at each half period, i.e. for each *k*. By (i) considering that the potential energy is maximum at these extremal positions of the lens and zero at the center and (ii) neglecting the variation of total mechanical energy (the sum of the kinetic and potential energy) over half a period, we can approximate the potential energy $E_{p}(|x_{\max}^{k}|)$ as $\frac{1}{2}(E_{c}^{k-}+E_{c}^{k+})$, where $E_{c}^{k-}$ and $E_{c}^{k+}$ correspond to the kinetic energy of the lens at the central position preceding and following its passage at the extremum $x_{\max}^{k}$. The results of these measurements are compared to the predictions in Fig. 4b for many different experiments. Then, from these curves, we deduce the value of the effective stiffness *Γ*, which corresponds to the curvature of the potential well $E_{p}(x_{0})$ and compare it to the theoretical value (Fig. 4c). Finally, by fitting the damped oscillator curves such as the one represented on Fig. 3b, we determine the damping coefficient (consistently observed around $2\alpha =\frac{1}{3}\;s^{-1}$), as well as the pseudopulsation of the oscillator, and compare the latter to its theoretical value $\omega =\sqrt{\omega_{0}^{2}-\alpha^{2}}$ (Fig. 4d). All these figures show quantitative agreement, meaning that the main physics is well captured by our model. Note that from a hydrodynamical standpoint, choosing a drag force varying linearly with the lens velocity (Stokes drag) means that the primary resistance to the lens motion originates from a laminar flow. In order to identify the primary resistance to the lens motion of typical velocity $U_{l}$, we examine the flows in the air and in the soap film. The shear in the air (dynamical viscosity $\mu_{\mathrm{air}}$) is established on a typical length equal to the radius of the lens $r_{l}≃6$ mm leading to a viscous stress of the order $∼\mu_{\mathrm{air}}\;U_{l}/r_{l}$ applied on the top and bottom surfaces of the lens, of order $r_{l}^{2}$, in contact with air. Therefore, the viscous drag on the lens from the air is of order $∼\mu_{\mathrm{air}}\;U_{l}\;r_{l}$. In the soap film, it was shown in similar systems that interfaces are mobile with such sodium dodecyl sulfate (surfactant) concentration (24, 25), leading to a velocity field induced by the motion of the lens dominated by in-plane flows, and invariant along the thickness of the soap film. The analysis of the viscous friction associated with the in-plane flow shows that a 2D apparent viscosity term appears (26, 27): $\mu_{2D}=2\mu_{s}+\mu \xi$, as the sum of (i) the surface viscosity $\mu_{s}$ associated with the shearing of the 2D surfactant loaded layers at the two liquid–air interfaces and (ii) the liquid bulk viscosity *μ* integrated over the thickness *ξ* of the film, μξ. As the in plane shear in the soap film occurs over a distance $r_{l}$, the 2D shear stress $∼\mu_{2D}\;U_{l}/r_{l}$ exerted on the contour of the lens $2\pi \;r_{l}$ leads to a drag force from the soap film on the lens of the order $∼\mu_{2D}\;U_{l}$. In order to determine the dominant source of viscous dissipation in the system we can assess the Boussinesq number that compares surface and subphase viscous drag forces $B_{o}=\mu_{2D}/\mu_{air}\;r_{l}=(2\mu_{s}+\mu \xi )/\mu_{air}\;r_{l}$. Measuring $\mu_{s}$ is a delicate matter and only an upper bound can be determined $\mu_{s}<$0.01 μPa·s·m (28). Our experimental parameters, ξ≃15  μm and $r_{l}≃6$ mm, and $\mu_{s}∼$0.01 μPa·s·m, leads to a moderate value of the Boussinesq number $B_{o}≲0.4$. This shows that the viscous drag exerted by the air phase is either dominant or of the same order of magnitude as the surface viscous stresses exerted by the film. The Reynolds number associated with the shear flow in the surrounding air writes Re=ρairrlUl/μair, with, $\rho_{\mathrm{air}}$ the density of air. We evaluate the characteristic velocity $U_{l}$ as the product of the amplitude (2 cm) of the oscillation and the frequency *f* (f=ω/2π=0.47 s${\;}^{-1}$ with the pulsation ω≃ 3 rad/s), $U_{l}≃$ 1 cm/s, leading to Re=4. The relatively low value of the Reynolds number and the good matching of the experimental lens dynamics with our linear model both suggest that the damping term is essentially linear.

Note also that a surface tension gradient may appear due to the motion of the lens. As the lens moves along the soap film, surfactant may accumulate on the interface at the front of the lens and be depleted at the rear. This effect induces a surface tension gradient along the lens, oriented opposite to its direction of motion, which can lead to additional drag on the lens (29).

### Orbiting single lens

**Figure 5:**
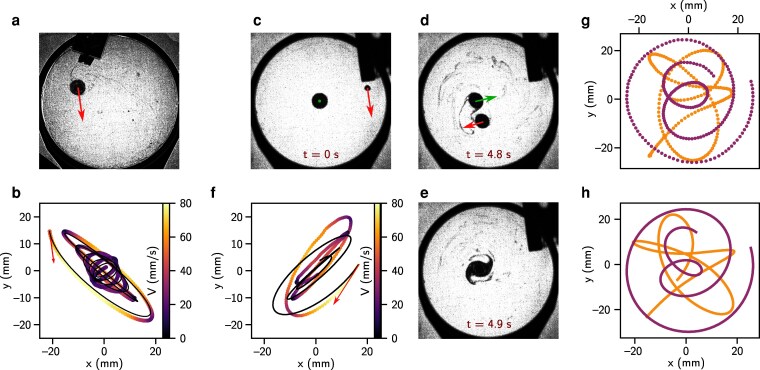
Orbits and merging of lenses. A single 28 mg lens is given an initial horizontal speed (red arrow) in the configuration shown in (a), resulting the trajectory plotted in (b), (the color denotes the speed magnitude) and shown in Movie S3. We superimpose this experimental trajectory with a simulated one (black line) based on the model from Eq. 15 with two oscillators of slightly different pulses $\omega_{x}$ and $\omega_{y}$ (the relative difference is about 8% between the two values). This anisotropy causes a shift in the direction of the orbit during the lens evolution. c–e) The evolution of two lenses of 35 mg orbiting and merging in a spiral structure before collapsing to form a new lens (not shown). On c and d), the two arrows indicate the speeds of the lenses (not to scale), with a zero initial speed for the central lens in (c). The trajectory of the barycenter of this system is plotted in (f) with the same conventions as in (b), along with a black line model for a lens of mass also 35 mg. g) Experimental trajectories of both lenses (m=35 mg). h) Numerical resolution of the dynamics following (Eq. 19).

Now let us expand the motion of the lens to one additional dimension, by considering its trajectory when it is injected with an initial horizontal velocity using the slide, as in Fig. 5a . An example of such trajectory is represented on Fig. 5b and Movie S3. To study it, we need to use Cartesian coordinates with axes $e_{x}$ and $e_{y}$, defined along the camera’s principal axes for the sake of simplicity and with the center of the soap film as the origin. The potential energy from Eq. 14 can of course be easily generalized in 2D as: $E_{p}(x_{0},y_{0})=\frac{1}{2}\Gamma r_{0}^{2}=\frac{1}{2}\Gamma (x_{0}^{2}+y_{0}^{2})$ since the potential energy only relies on the distance $r_{0}$ from the center. It can then be used to develop Newton’s second law, and project it onto the two orthogonal directions (Ox) and (Oy). It appears that since the potential energy of the system varies quadratically with the distance separating the lens from the soap film center, the equations of motion in each direction are decoupled and governed by damped harmonic oscillator dynamics. The 2D motion of the lens is therefore formalized by two orthogonal and decoupled harmonic oscillators whose solutions read:


(15) $$\left\{ \begin{array}{c} x_{0}(t)\;=e^{-\alpha t}\;\left[x_{i}\cos\;(\omega_{x}t)+\frac{v_{xi}+\alpha x_{i}}{\omega_{x}}\sin\;(\omega_{x}t)\right]\\ y_{0}(t)\;=e^{-\alpha t}\;\left[y_{i}\cos\;(\omega_{y}t)+\frac{v_{yi}+\alpha y_{i}}{\omega_{y}}\sin\;(\omega_{y}t)\right]. \end{array}\right.$$

with $x_{i}$, $y_{i}$, $v_{xi}$, and $v_{yi}$ the initial coordinate and velocity of the lens along *x* and *y* directions, respectively and $\omega_{x}$ and $\omega_{y}$ the eigenfrequencies in both directions.

For a perfectly isotropic experiment, the natural frequencies $\omega_{x}$ and $\omega_{y}$ would be both equal to *ω* in Eq. 15, leading to an elliptical spiral trajectory toward the center. However, Fig. 5b and Movie S3 show that the lens exhibits a complex trajectory progressively drifting toward a straight trajectory before reversing its orbital rotation direction. This behavior can be rationalized by introducing a slight anisotropy of the soap film leading to $\omega_{x}=\omega +\delta \omega$ and $\omega_{y}=\omega -\delta \omega$, which induces a progressive drift between the two orthogonal oscillators. By introducing this small anisotropy under the form of δω≃0.04ω, we see that the model nicely recovers the experimental trajectory, plotted in black on Fig. 5b. The cause of this anisotropy is yet to be rationalized but could be explained with inhomogeneities in the soap film thickness or a slight tilt of the frame that prevents perfect horizontality: a well-known anisotropic effect observed on the support for Foucault's pendulum, first described by Kamerlingh Onnes (30), that induces Lissajoux figures damped here by viscosity.

### Two orbiting lenses

Now, we can dive into the richest part of this work: the dynamics of two orbiting lenses. A second droplet of water is injected with a nonzero tangential speed around a resting central lens, both having the same masses to begin with. This causes the two bodies to orbit each other for some revolutions and finally to collide and merge (see Fig. 5c–e and Movie S4). Before the collision, the barycenter of the two lenses is expected to follow the same trajectory as a single lens. If we track this barycenter position, and compare it with the previous model with a mass *m* (the arithmetic mean of the two interacting masses), we indeed obtain a good agreement between our prediction and the experiments, see Fig. 5f. However, resolving the relative motion of the two lenses, as shown in Fig. 5g, pertains to solving a three-body problem, consisting of each lens and the central force exerted by the supporting film. Hence, it is necessary to add the pairwise attraction force between the two lenses. This force derives from an energy of interaction $E_{\mathrm{pair}}$ identified as the gravitational potential energy of a given lens $L_{j}$ into the deflexion of the film caused by the other one $\left(L_{i}\right)$. Following Nicholson (5), we assume that when two lenses are deposited on the soap film, the total interfacial deformation is the sum of the profiles around the isolated lenses on the soap film (linear superposition approximation). Identifying the interaction pair energy as the product of the weight $m_{j}\;g$ of $L_{j}$ with its vertical displacement caused by the profile $z_{i}$ of the isolated other lens $L_{i}$ leads to (details in Supplementary material):


(16) $$E_{\mathrm{pair}}=m_{j}\;g\;z_{i}(r_{ij}),$$

where $r_{ij}$ is the horizontal distance between the two lenses, and the profile of an isolated lens on a weightless membrane $z_{i}(r_{ij})$ is equal to:


(17) $$z_{i}(r_{ij})=\frac{m_{i}\;g}{4\pi \sigma }\ln\;\left(\frac{r_{ij}}{R}\right),$$

with $m_{i}$ the mass of $L_{i}$. This simple expression is obtained within the approximation that $L_{i}$ and the studied point are both far away from the edge of the film, i.e. when $\left(r_{i}\ll R,r_{ij}\ll R\right)$, with $r_{i}$ the distance separating the center of the soap film from the center of the lens $L_{i}$. As long as these conditions are met—in fact the approximation appears to stand in experiments even for $r_{i}$ and $r_{ij}$ being as large as 0.6R—we can derive the force driving the second lens $L_{j}$ toward $L_{i}$: $F_{\mathrm{pair}}=-\nabla (E_{\mathrm{pair}})$, i.e.:


(18) $$F_{\mathrm{pair}}=-\frac{g^{2}}{4\pi \sigma }\frac{m_{i}\;m_{j}}{r_{ij}}\;e_{ij},$$

$e_{ij}$ being the unit vector between $L_{i}$ and $L_{j}$. Adding this Newton-like force in 2D (see Ref. (31) for a discussion on the distance dependence of Newtonian gravity with the spatial dimension, here in $1/r^{\left(2-1\right)}=1/r$) to Eq. 13 yields the following dynamics equations driving a lens *i* pulled by the other lens *j*, in any set of Cartesian coordinates (x,y) centered on the film:


(19) $$\left(\begin{array}{c} {\ddot{x}}_{i}\\ {\ddot{y}}_{i} \end{array}\right)+\frac{\Gamma_{i}}{m_{i}}\left(\begin{array}{c} x_{i}\\ y_{i} \end{array}\right)+\frac{T_{ij}}{m_{i}}\cdot \left(\begin{array}{c} x_{i}-x_{j}\\ y_{i}-y_{j} \end{array}\right)+2\alpha \left(\begin{array}{c} {\dot{x}}_{i}\\ {\dot{y}}_{i} \end{array}\right)=\left(\begin{array}{c} 0\\ 0 \end{array}\right),$$

where $\Gamma_{i}$ is the stiffness of the potential well from Eq. 14 applied to the lens *i*:


$$\Gamma_{i}=m_{i}\;g\left(\frac{\xi }{4\ell_{c}^{2}}+\frac{m_{i}g}{4\pi \sigma R^{2}}\right),$$

and


$$T_{ij}=\frac{g^{2}}{4\pi \sigma }\frac{m_{i}\;m_{j}}{r_{ij}^{2}}\;.$$

Due to the coupling between the dynamics of the two lenses (see Eq. 19), the resolution of the system dynamics was performed numerically and compared to experimentally trajectories, see Fig. 5g and h. The results show good agreement with slightly adjusted values of the friction coefficient ($2\alpha =0.32\;s^{-1}$) and film thickness (ξ=20μm), compared to the values measured during experiments with one lens. It is worth noting that in those simulations, the lenses did go further than 0.4R from the center, which could also cause some discrepancies between the experiments and simulations.

### Lenses merging

After orbiting, the lenses end up merging into a spiral shape, shown in Fig. 5e, which, then, collapses to form a new circular lens. Tidal and centrifugal forces are suspected to drive the deformation of the lenses in close proximity of each other, stretching them into the arms of a spiral. For lenses of different masses, we observe that a light body injected with an initial speed will experience far greater deformations than the heavy one it orbits around (see Movie S5). Precise description and modeling of these short-range interactions and deformations, as well as predicting the final spiral shape, are still work in progress and outside the scope of this article.

## Discussion

**Figure 6:**
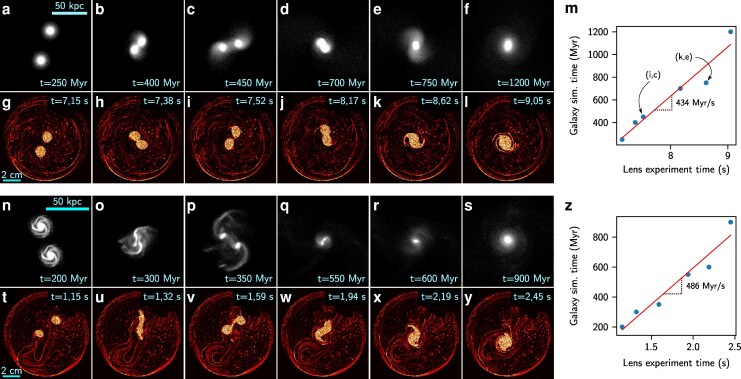
Comparison between simulations of coplanar merging galaxies and dynamics of lenses merging on a soap film. a–f and n–s) are sequences of merging galaxies from the GalMer database (32), respectively featuring two giant elliptical galaxies and two giant spiral galaxies. g–l and t–y) are two merging sequences of lenses of same masses (see Movies S6 and S7, respectively), respectively 28 and 18 mg, processed by the Schlieren software ComBOS (21). m and z) Correspondence between the timescales of the galaxies simulations (in Myr) and the lens experiments (in s) for each sequence. Each point refers to a couple of images, e.g. i and c, or k and e in m).

In this work, we have demonstrated that: (i) a droplet deposited at the center of a soap film induces a deformation (a “lens”) with a universal radius independent of the lens mass; (ii) when a lens is placed off-center, it experiences a central restoring force leading to harmonic motion in the absence of initial azimuthal velocity, or a orbital dynamics otherwise; and (iii) the intricate motion of two lenses orbiting on a soap film is governed by a combination of this harmonic potential well and a pair interaction force $F_{\mathrm{pair}}=-G_{2D}\frac{m_{i}\;m_{j}}{r_{ij}}\;e_{ij}$, with $G_{2D}=g^{2}/4\pi \sigma$, which is essentially a reduced-dimensional (2D) version of Newton’s law of universal gravitation (31, 33). These effects emerge from the weak deformation of the supporting film, in a manner reminiscent of how Newtonian gravity arises as a weak-field approximation of General Relativity, where gravity is described as the curvature of spacetime. Strikingly, the dynamics and fusion of two identical massive and light lenses mirror the simulated mergers of giant elliptical and spiral coplanar galaxies, respectively (Fig. 6a–l and n-y), albeit at vastly reduced time (and length) scales, with a scaling correspondence of typically 460Myr/s as shown in the relations between galaxy simulation time and lens experiment time (Fig. 6m and z). Furthermore, this lens coalescence sequence produces structures (Fig. 1d–i) that bear a remarkable resemblance to those observed in coplanar galaxy mergers.

While this analogy remains in its early stages and possesses inherent limitations—such as the presence of relatively strong dissipative mechanisms and its restriction to planar dynamics—the emergence of a Newton-like law of lens attraction, tidal interactions, and complex merging structures suggests a compelling parallel between these human-scale fluidic experiments and large-scale astrophysical processes, such as galactic fusion. This unexpected bridge between soft matter physics and astrophysics opens perspective for investigating, on a human time scale, simplified representations of phenomena that occur on time scales beyond the reach of human observation.

## Supplementary Material

pgag079_Supplementary_Data

## Data Availability

All experimental data and analysis code underlying the findings of this study are publicly available on Zenodo data repository, https://doi.org/10.5281/zenodo.19039715.

## Preprints

This manuscript was posted on a preprint site: https://doi.org/10.48550/arXiv.2503.08331.
