# Supplementary material for: Orbiting, colliding, and merging liquid lenses on a soap film: Toward gravitational analogs
Source: PNAS Nexus. 2026 Mar 24;5(4):pgag079. doi: 10.1093/pnasnexus/pgag079 (PMC13108600; doi:10.1093/pnasnexus/pgag079)
Supplement: pgag079_Supplementary_Data [file pgag079_supplementary_data.zip › PNASNEXUS-PNASNEXUS-2025-01772-T-s08.pdf]

Supporting Information for

Orbiting, colliding and merging liquid lenses on a soap film: toward gravitational analogues

J.-P. Martischang, B. Reichert, I. Haouche, G. Rousseaux, A. Duchesne, M. Baudoin

Michael Baudoin, Benjamin Reichert
E-mails: michael.baudoin@univ-lille.fr benjamin.reichert@univ-lille.fr

This PDF file includes:

Supporting text
Figs. S1 to S4
Legends for Movies S1 to S7
SI References

Other supporting materials for this manuscript include the following:

Movies S1 to S7

Supporting Information Text

1. Equilibrium of a membrane portion

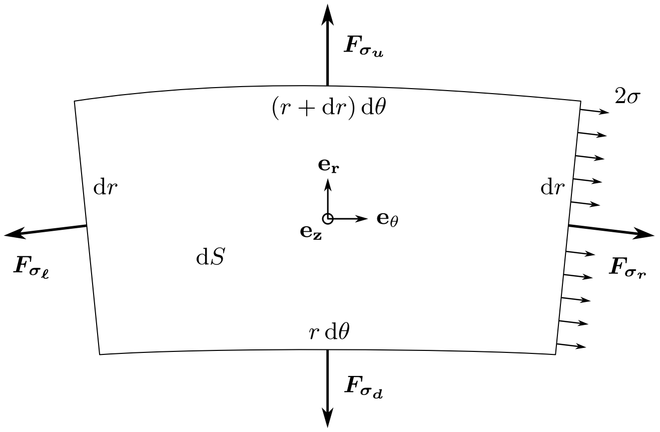

Fig. S1. Equilibrium of a portion of the soap film of surface $\mathrm{d}S$, at distance r from the center, over the radius $\mathrm{d}r$ and the angle $\mathrm{d}\theta$. Surface tension 2σ applies on the four sides of the elementary portion, generating the tension forces $\mathbf{F}_{\sigma_u}$, $\mathbf{F}_{\sigma_d}$, $\mathbf{F}_{\sigma_\ell}$ and $\mathbf{F}_{\sigma_r}$.

The system is parametrized in cylindrical coordinates (r, θ, z), with the origin O at the center of the film. The profile of the soap film is deduced from the force balance on an elementary portion of area $\mathrm{dS} = \mathrm{r\,dr\,d}\theta$ of the soap membrane (Fig. S1). The equilibrium of the fluidic membrane is governed by three forces; its weight denoted $\mathbf{P} = -\rho\, g\, \xi\, \mathrm{dS}\, \mathbf{e_z}$, with ρ the density of the liquid, g the gravitational acceleration, and ξ the thickness of the film, a load $-Q(\mathbf{r})\mathrm{dS}\, \mathbf{e_z}$ of distribution $Q(\mathbf{r})$ hanging on the membrane, and capillary forces exerted on each side of the portion dS of a membrane of tension 2σ,

$$\begin{cases} \mathbf{F}_{\sigma_d} & = 2\sigma \cdot r\,\mathrm{d}\theta\,(-\cos\alpha\,\mathbf{e_r} - \sin\alpha\,\mathbf{e_z})|_r \\ \mathbf{F}_{\sigma_u} & = 2\sigma \cdot (r + \mathrm{d}r)\,\mathrm{d}\theta\,(\cos\alpha\,\mathbf{e_r} + \sin\alpha\,\mathbf{e_z})|_{r+\mathrm{d}r} \\ \mathbf{F}_{\sigma_\ell} & = 2\sigma \cdot \mathrm{d}r\,(-\cos\chi\,\mathbf{e_\theta} - \sin\chi\,\mathbf{e_z})|_\theta \\ \mathbf{F}_{\sigma_r} & = 2\sigma \cdot r\,\mathrm{d}r\,(\cos\chi\,\mathbf{e_\theta} + \sin\chi\,\mathbf{e_z})|_{\theta+\mathrm{d}\theta} \end{cases}.$$

where the inclination of the film portion is parametrized by two angles: α around the axis $\mathbf{e_\theta}$ such as $\tan\alpha = \mathrm{d}z/\mathrm{d}r$, and χ around $\mathbf{e_r}$ with $\tan\chi = \mathrm{d}z/(r\mathrm{d}\theta)$.

In the small angles approximation ($\sin\alpha \simeq \tan\alpha$, $\sin\chi \simeq \tan\chi$), the projection of the force balance in the vertical direction gives

$$2\sigma \left[\frac{\partial^2 z}{\partial r^2} + \frac{1}{r}\frac{\partial z}{\partial r} + \frac{1}{r^2}\frac{\partial^2 z}{\partial \theta^2}\right] \mathrm{d}S - \rho\, g\, \xi\, \mathrm{d}S = 0, \quad [1]$$

leading to

$$2\sigma\Delta z = \rho\, g\, \xi + Q(\mathbf{r}). \quad [2]$$

This Poisson equation shows that the warping of the membrane, of curvature Δz, is induced by two distinct sources, the membrane weight and the external load of distribution $Q(\mathbf{r})$. In the case of a single lens hanging on the membrane at location $\mathbf{r}_0$, this load is punctual and its magnitude is identified as the weight mg of the lens. The load distribution simply becomes $Q(\mathbf{r}) = mg\,\delta(\mathbf{r} - \mathbf{r}_0)$, with δ being Dirac's delta function.

2. Equilibrium profile of the soap film

The equilibrium profile of the membrane for a lens located at $\mathbf{r}_0 = (r_0, \theta_0)$ in cylindrical coordinate, is obtained from the following pressure balance

$$2\sigma\Delta z(\mathbf{r}, \mathbf{r_0}) = \rho\, g\, \xi + m\, g\, \delta(\mathbf{r} - \mathbf{r}_0). \quad [3]$$

Since this equation is linear, the equilibrium profile of the membrane $z(\mathbf{r}, \mathbf{r}_0)$ is sought as the superposition of two profiles, each stemming from a contribution in the rhs member of the pressure balance Eq. (3), a parabola $z_p(\mathbf{r})$ induced by the membrane weight, and a catenoid $z_c(\mathbf{r}, \mathbf{r}_0)$ due to the punctual load:

$$z(\mathbf{r}, \mathbf{r_0}) = z_p(\mathbf{r}) + z_c(\mathbf{r}, \mathbf{r}_0). \quad [4]$$

The profile of a weighing soap film free from any load is axisymmetric and is obtained from the resolution of

$$\Delta z_p = \frac{\rho g \xi}{2\sigma} \quad [5]$$

as a parabola:

$$z_p(\mathbf{r}) = \frac{\xi}{8\ell_c^2}(r^2 - R^2). \qquad [6]$$

The profile of a weightless membrane with a lens positioned at $\mathbf{r}_0$ results from the resolution of the Poisson equation

$$\Delta z_c = \frac{mg}{2\sigma}\delta(\mathbf{r} - \mathbf{r}_0). \qquad [7]$$

and is a catenoid:

$$z_c(\mathbf{r}, \mathbf{r_0}) = \frac{mg}{2\sigma} G(\mathbf{r}, \mathbf{r}_0) \qquad [8]$$

where G is Green's function for problem Eq. (7) on the disc of radius R, satisfying the condition $\Delta G(\mathbf{r}, \mathbf{r}_0) = \delta(\mathbf{r} - \mathbf{r}_0)$, with the boundary condition $G(\mathbf{r}, \mathbf{r}_0) = 0$ for $|\mathbf{r}| = R$. In other terms:

$$G(\mathbf{r}, \mathbf{r}_0) = \frac{1}{4\pi} \ln \left[\frac{r^2 + r_0^2 - 2\, r\, r_0 \cos(\theta - \theta_0)}{R^2 + r^2 r_0^2 / R^2 - 2\, r\, r_0 \cos(\theta - \theta_0)} \right].$$

The general expression of the equilibrium profile of the soap film eventually writes:

$$\begin{aligned} z(\mathbf{r}, \mathbf{r}_0) = {} & \frac{\xi}{8\ell_c^2}(r^2 - R^2) + \\ & \frac{mg}{8\pi\sigma} \ln \left[\frac{r^2 + r_0^2 - 2\, r\, r_0 \cos(\theta - \theta_0)}{R^2 + r^2 r_0^2 / R^2 - 2\, r\, r_0 \cos(\theta - \theta_0)} \right]. \end{aligned} \qquad [9]$$

In the case of a lens lying at the center of the soap film ($\mathbf{r}_0 = \mathbf{0}$), the profile of the soap film becomes axisymmetric and simplifies into:

$$z(r) = \frac{\xi}{8\ell_c^2}(r^2 - R^2) + \frac{mg}{4\pi\sigma} \ln\left(\frac{r}{R}\right). \qquad [10]$$

This axisymmetric profile is displayed in Fig.2b in the main text.

3. Equilibrium profile of the lens

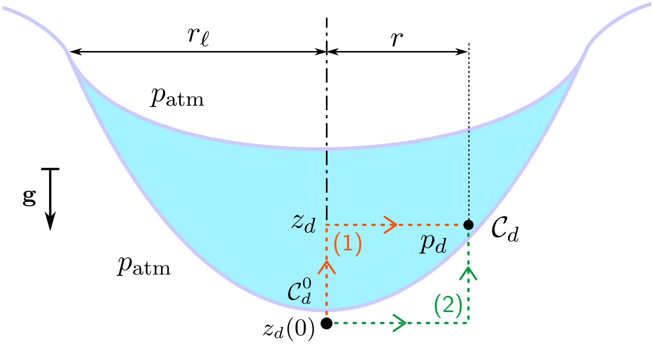

Fig. S2. Illustration of two possible path (1) and (2) to evaluate the pressure $p_d(r)$ at the altitude $z_d(r)$, starting from a central point just under the bottom of the film, at $z_d(0)$.

In this part, the lens is at rest at the center of the film. Due to axisymmetry, the coordinates of any point will be reduced to the radial one, r. The equilibrium profile of the thick region of the liquid phase ($r \in [0, r_\ell]$) is determined in the following. Using the notations defined in Fig.2a in the main text, we first consider two Laplace pressure jumps at the upper and lower interfaces

$$\begin{cases} p_{\text{atm}} - p_u(r) = \sigma\, \mathcal{C}_u(r) \\ p_d(r) - p_{\text{atm}} = \sigma\, \mathcal{C}_d(r) \end{cases} \qquad [11]$$

where $\mathcal{C}_u$ and $\mathcal{C}_d$ are the upper and lower interfacial curvatures, and σ the surface tension of the liquid phase, supposed equal to that of the soap film. Besides, the hydrostatic pressure balance in the liquid phase gives:

$$p_d(r) = p_u(r) + \rho\, g\, h(r) \qquad [12]$$

with $h(r)$ the thickness of the liquid phase. By combining eq. 11 and 12, we can write:

$$\mathcal{C}_u(r) + \mathcal{C}_d(r) = \frac{h(r)}{\ell_c^2}, \qquad [13]$$

where $\ell_c^2 = \frac{\sigma}{\rho g}$ is the squared capillary length. This writes, in a more detailed fashion:

$$\frac{\partial^2 z_u}{\partial r^2} + \frac{1}{r}\frac{\partial z_u}{\partial r} - \frac{z_u}{\ell_c^2} = -\frac{\partial^2 z_d}{\partial r^2} - \frac{1}{r}\frac{\partial z_d}{\partial r} - \frac{z_d}{\ell_c^2}\,. \quad [14]$$

Another equation can help us divide this one in two parts. Let's look at the figure S2. Starting from a point just under the lowest point of the film (altitude $z_d(0)$, we can calculate the pressure just above the lower interface for a given radius r, $p_d(r)$, either by going up, then right (path 1) or right, then up (path 2) in the convention of the figure. The first case gives us:

$$p_d(r) = p_{\mathrm{atm}} + \sigma \mathcal{C}_d^0 + \rho g\left[z_d(r) - z_d(0)\right] \quad [15]$$

where $\mathcal{C}_d^0$ is the curvature of the lower interface at the center of the film; while the second one yields:

$$p_d(r) = p_{\mathrm{atm}} + \sigma \mathcal{C}_d(r). \quad [16]$$

We can then write:

$$\mathcal{C}_d^0 - \frac{z_d(r) - z_d(0)}{\ell_c^2} = \mathcal{C}_d(r) \quad [17]$$

which gives:

$$\frac{\partial^2 z_d}{\partial r^2} + \frac{1}{r}\frac{\partial z_d}{\partial r} + \frac{z_d}{\ell_c^2} = \mathcal{C}_d^0 + \frac{z_d(0)}{\ell_c^2}. \quad [18]$$

If we name the constant $C = \mathcal{C}_d^0 + \frac{z_d(0)}{\ell_c^2}$, we may split equation 14 in two:

$$\begin{cases} \dfrac{\partial^2 z_u}{\partial r^2} + \dfrac{1}{r}\dfrac{\partial z_u}{\partial r} - \dfrac{z_u}{\ell_c^2} = -C \\ \dfrac{\partial^2 z_d}{\partial r^2} + \dfrac{1}{r}\dfrac{\partial z_d}{\partial r} + \dfrac{z_d}{\ell_c^2} = C \end{cases}. \quad [19]$$

These are two Bessel equations of zeroth order. Avoiding divergence at $r = 0$, we get the solutions:

$$\begin{cases} z_u(r) = M \cdot \mathrm{I}_0(\frac{r}{\ell_c}) + \ell_c^2 C \\ z_d(r) = L \cdot \mathrm{J}_0(\frac{r}{\ell_c}) + \ell_c^2 C \end{cases} \quad [20]$$

where $M \in \mathbb{R}$, $L \in \mathbb{R}$, and J_0 and I_0 are Bessel and modified Bessel functions of zeroth order.

The film and the liquid of the lens being miscible, there is no triple line in $r = r_\ell$, so no reason for a discontinuity in the profile derivatives. We can therefore join z_u, z_d and z at r_ℓ with both their values and derivatives. From $\frac{\partial z}{\partial r} = \frac{\partial z_u}{\partial r}$, we get, defining $\eta = r_\ell/\ell_c$:

$$M = \frac{1}{\mathrm{I}_1(\eta)} \cdot \left(\frac{mg}{4\pi\sigma\eta} + \frac{\xi\eta}{4}\right) \quad [21]$$

Then, from $z_u(r_\ell) = z_d(r_\ell)$, we get:

$$L = \frac{\mathrm{I}_0(\eta)}{\mathrm{J}_0(\eta)} M. \quad [22]$$

Using this and the fact that $\frac{\partial z_u}{\partial r} = \frac{\partial z_d}{\partial r}$, we can then write:

$$\frac{\mathrm{I}_0(\eta)}{\mathrm{I}_1(\eta)} = -\frac{\mathrm{J}_0(\eta)}{\mathrm{J}_1(\eta)} \quad [23]$$

with J_1 and I_1 being Bessel and modified Bessel functions of first order. This gives an expression to numerically determine η, from which we get r_ℓ. Notice that with this, r_ℓ only depends on the capillary length ℓ_c of the lens, and not on its mass itself.

The last raccording to do is $z(r_\ell) = z_u(r_\ell)$, or:

$$\frac{mg}{4\pi\sigma}\ln\left(\frac{r_\ell}{R}\right) + \frac{\xi}{8\ell_c^2}(r_\ell^2 - R^2) = M \cdot \mathrm{I}_0(\eta) + \ell_c^2 C \quad [24]$$

from which we get, using equations 21 and 23:

$$\ell_c^2 C = \frac{\mathrm{J}_0(\eta)}{\mathrm{J}_1(\eta)}\left(\frac{mg}{4\pi\sigma\eta} + \frac{\xi\eta}{4}\right) + z(r_\ell). \quad [25]$$

After injecting equation Eq. (25) into equations Eq. (20), we finally get:

$$\begin{cases} z_u(r) = \frac{\mathrm{J}_0(\eta)}{\mathrm{J}_1(\eta)}\left(\frac{mg}{4\pi\sigma\eta} + \frac{\xi\eta}{4}\right)\left(1 - \frac{\mathrm{I}_0(r/\ell_c)}{\mathrm{I}_0(\eta)}\right) + z(r_\ell) \\ z_d(r) = \frac{\mathrm{J}_0(\eta)}{\mathrm{J}_1(\eta)}\left(\frac{mg}{4\pi\sigma\eta} + \frac{\xi\eta}{4}\right)\left(1 - \frac{\mathrm{J}_0(r/\ell_c)}{\mathrm{J}_0(\eta)}\right) + z(r_\ell) \end{cases}. \quad [26]$$

4. Trajectory of the lens

Here, we suppose that the "in plane" – the plane being $(O, \mathbf{e_r}, \mathbf{e_\theta})$ – trajectory of the lens is a straight line, confined to a diameter of the soap film, $\theta_0 \equiv \text{cst}\,[\pi]$. The trajectory of the lens is obtained from the expression of the membrane equilibrium profile $z(\mathbf{r}, \mathbf{r}_0) = z_p(\mathbf{r}) + z_c(\mathbf{r}, \mathbf{r}_0)$ (see equations Eq. (4) and Eq. (9)) evaluated at $\mathbf{r} = \mathbf{r}_0$ ($r = r_0$ and $\theta = \theta_0$) where the lens center lies. The evaluation of the parabolic contribution to the trajectory is straightforward and gives

$$z_p(\mathbf{r} = \mathbf{r}_0) = \frac{\xi}{8\ell_c^2}(r_0^2 - R^2). \qquad [27]$$

The evaluation of the catenoidal contribution to the trajectory is more involved since the catenoid $z_c(\mathbf{r}, \mathbf{r}_0)$ diverges at $\mathbf{r} = \mathbf{r}_0$. The expression of the catenoid only has a physical meaning outside the lens, i.e. for $|\mathbf{r} - \mathbf{r}_0| > r_\ell$. Therefore, the vertical position of the center of the lens on this catenoid is defined as the average of the catenoid altitudes on opposite sides of the lens, on $r = r_0 - r_\ell$ and $r = r_0 + r_\ell$:

$$z_c(\mathbf{r} = \mathbf{r_0}, \mathbf{r}_0) = < z_c >= \frac{1}{2}[z_c(r_0 - r_\ell) + z_c(r_0 + r_\ell)], \qquad [28]$$

which writes:

$$< z_c >= \frac{mg}{8\pi\sigma}\left\{2\ln\left(\frac{r_\ell}{R}\right) - \ln\left[\left(1 - \frac{r_0^2}{R^2}\right)^2 - \frac{r_0^2}{R^2}\frac{r_\ell^2}{R^2}\right]\right\}. \qquad [29]$$

In the limit of a small lens ($r_\ell \ll R$) oscillating in the vicinity of the center of the soap film ($r_0 \ll R$), the altitude of the catenoid at $\mathbf{r} = \mathbf{r}_0$ simplifies into

$$< z_c >= \frac{mg}{4\pi\sigma}\left[\ln\left(\frac{r_\ell}{R}\right) + \left(\frac{r_0}{R}\right)^2\right]. \qquad [30]$$

and the expression of the trajectory $z_0(r_0) = z(\mathbf{r}_0, \mathbf{r}_0)$ becomes:

$$z_0(r_0) = \left(\frac{\xi}{8\ell_c^2} + \frac{mg}{4\pi\sigma R^2}\right) r_0^2 + \frac{mg}{4\pi\sigma}\ln\left(\frac{r_\ell}{R}\right) - \frac{\xi R^2}{8\ell_c^2}. \qquad [31]$$

5. Energy of the system

We aim here to obtain the potential energy from which derives the capillary force attracting the lens towards the center of the film. The motion of the lens in the horizontal plane $(O, \mathbf{e_r}, \mathbf{e_\theta})$ of the soap film is assumed to be a straight line along its diameter.

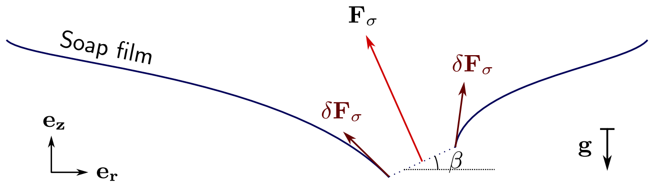

Fig. S3. Exaggerated representation of the tilted lens on the soap film, with the capillary forces $\delta\mathbf{F}_\sigma$ exerted on the contour of the body and their sum $\mathbf{F}_\sigma$. The lens is tilted with an angle β with respect to the horizontal axis.

In order to derive the capillary force acting on the lens in this plane, we first need to consider a force balance in the plane containing the axis of motion and the vertical direction, namely the $(O, \mathbf{e_r}, \mathbf{e_z})$ plane. The two forces acting on the lens in this plane are the weight of the lens $-mg\,\mathbf{e_z}$ and the surface tension force $\mathbf{F}_\sigma = \int_\mathcal{C} \delta\boldsymbol{F_\sigma}$, where $||\delta\boldsymbol{F_\sigma}|| = 2\sigma\,\mathrm{d}\ell$, and $\mathcal{C}$ is the lens contour (see Fig. S3). Since the inertia of the lens is neglected in the vertical direction, the lens is in equilibrium in the z direction and the weight of the lens is balanced by the surface tension force:

$$\mathbf{F}_\sigma \cdot \mathbf{e_z} - mg = 0$$

This vertical equilibrium can be recast into an explicit expression by introducing the tilt angle of the lens β,

$$||\mathbf{F}_\sigma||\cos\beta - mg = 0. \qquad [32]$$

The central capillary force attracting any body on the soap film towards its center identifies as the projection of $\mathbf{F}_\sigma$ onto the horizontal plane

$$F_{\sigma r} = \mathbf{F}_\sigma \cdot \mathbf{e_r} = -||\mathbf{F}_\sigma||\sin\beta = -mg\tan\beta. \qquad [33]$$

The horizontal capillary restoring force is therefore identified as the gravitational force exerted on a lens in an effective slope $\tan\beta$. Based on the equilibrium shape of the soap membrane, the effective slope has two contributions: the slope due to the parabolic shape of a weighing membrane $\frac{\mathrm{d}z_p}{\mathrm{d}r}(r=r_o)$ and a net slope due to the slope difference of the catenoid on opposite sides of the lens $< z_c^{'} >= \left[\frac{\partial z_c}{\partial r}(r=r_0+r_\ell)+\frac{\partial z_c}{\partial r}(r=r_0-r_\ell)\right]/2$,

$$\tan\beta = \left.\frac{\mathrm{d}z_p}{\mathrm{d}r}\right|_{r=r_0} + < z_c^{'} > \qquad [34]$$

An explicit expression for the net slope is derived from the membrane equilibrium profile (equations Eq. (6) and Eq. (8)):

$$\tan\beta = \frac{\xi r_0}{4\ell_c^2} + \frac{m\,g}{4\,R\,\pi\sigma}\left[\frac{r_o/R - r_o^3/R^3}{(1-r_o^2/R^2)^2-(r_o\,r_\ell/R^2)^2}\right]. \qquad [35]$$

In the limit of a small lens ($r_\ell \ll R$) oscillating in the vicinity of the center of the soap film ($r_0 \ll R$), the capillary restoring force writes at first order in $\varepsilon = r_0/R$:

$$F_{\sigma r} = -\left[\frac{mg\xi}{4\ell_c^2} + \frac{(mg)^2}{4\pi\sigma R^2}\right] r_0 \qquad [36]$$

The potential energy E_p of this system composed of a lens hanging on a fluidic membrane is the potential energy deriving from the capillary restoring force $\mathrm{F}_{\sigma r} = -\partial \mathrm{E_p}/\partial \mathrm{r_0}$, i.e.:

$$E_p = mg\left(\frac{\xi}{8\ell_c^2} + \frac{mg}{8\pi\sigma R^2}\right) r_0^2. \qquad [37]$$

We see that the energy of the system varies quadratically with the position r_0 of the lens. It is minimal for a lens located at the center of the soap film, which therefore acts as a harmonic potential well.

6. Pair attraction between two lenses

We consider a soap film loaded with two lenses: one $\mathscr{L}_i$ of mass m_i, located at (r_i, θ_i), and another $\mathscr{L}_j$ of mass m_j, located at (r_j, θ_j). We want to determine the pair attraction force between $\mathscr{L}_i$ and $\mathscr{L}_j$, via the membrane deformation. The main assumption here is that the total deformation of the interface when two lenses are deposited on the soap film is the sum of the profiles each lens would generate if it was deposited alone. This is the linear superposition approximation Nicholson introduced to derive an analytical expression of the capillary force between two floating bubbles (1).

We start by computing the deformation of the film induced by $\mathscr{L}_i$ (see equation Eq. (8)), which will be expressed in a new frame centered on $\mathscr{L}_i$. We focus in particular on the deflexion of the membrane along the axis $(\mathscr{L}_i, \mathscr{L}_j)$. First, the warping $z_{\mathscr{L}_i}$ of the membrane at position (r,θ) caused by the lens $\mathscr{L}_i$ located at position (r_i, θ_i) is

$$z_{\mathscr{L}_i}(r,\theta) = \frac{m_i g}{8\pi\sigma}\left[2\ln\left(\frac{d}{R}\right) - \ln\left(1-2\varepsilon\cos(\theta-\theta_i)+\varepsilon^2\right)\right]. \qquad [38]$$

with $\varepsilon = r r_i/R^2$ and $d = ||\mathbf{r}-\mathbf{r}_i|| = \sqrt{r^2+r_i^2-2\,r\,r_i\,\cos(\theta-\theta_i)}$ the distance separating our study point from $\mathscr{L}_i$.

Here, we are especially interested in the the warping experienced by the lens $\mathscr{L}_j$, i.e. $z_{\mathscr{L}_i}(r_j, \theta_j)$. If we assume that both lenses are located in the immediate vicinity of the center of the soap film, then $r_i \ll R$, $r_j \ll R$ and $\varepsilon \ll 1$. We call r_{ij} the particular value of $d = ||\mathbf{r_j}-\mathbf{r_i}||$. Then, the first order approximation of the membrane deformation at the lens $\mathscr{L}_j$ writes:

$$z_{\mathscr{L}_i}(r_j,\theta_j) \simeq \frac{m_i\,g}{4\pi\sigma}\left[\ln\left(\frac{r_{ij}}{R}\right) + \varepsilon\cos(\theta_j-\theta_i)\right]. \qquad [39]$$

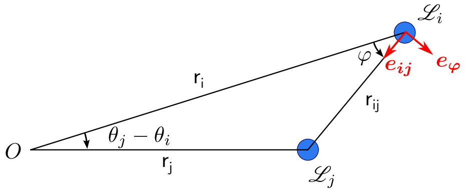

Fig. S4. Triangle formed by both lenses $\mathscr{L}_i$ and $\mathscr{L}_j$, and the center of the film O.

In order to recast the membrane deflexion profile (see equation Eq. (39)) into the frame centered on $\mathscr{L}_i$, the polar angle θ is replaced by an angle φ around $(\mathscr{L}_i, \mathbf{e_z})$ and the radial coordinate r_j is replaced by r_{ij} (see Fig. S4). The vector basis associated to this frame of reference is denoted $(\mathbf{e_{ij}}, \boldsymbol{e_\varphi})$. This change of variable is performed by applying twice Al-Kashi's theorem on the triangle in Fig. S4:

$$\cos(\theta_j-\theta_i) = \frac{r_i - r_{ij}\,\cos\varphi}{r_j}. \qquad [40]$$

and allows to determine the profile equation into the new coordinate system (d,φ):

$$z_{\mathscr{L}_i}(r_{ij},\varphi) = \frac{m_i\, g}{4\pi\sigma}\left[\ln\left(\frac{r_{ij}}{R}\right) + r_i\,\frac{r_i - r_{ij}\,\cos\varphi}{R^2}\right]\,. \qquad [41]$$

Having calculated the interfacial deflexion caused by $\mathscr{L}_i$, and knowing the weight of $\mathscr{L}_j$, we can now obtain the energy of interaction of the two lenses. This pair energy is the product of the weight of $\mathscr{L}_j$ with its vertical displacement along the membrane deformation induced by the presence of its counterpart $\mathscr{L}_i$:

$$E_{\text{pair}} = m_j\, g\, z_{\mathscr{L}_i} \qquad [42]$$

Once again, in this analysis, we essentially focus on lenses dynamics occurring in the immediate vicinity of the soap film center which implies $r_i \ll R$ and $r_{ij} \ll R$. At dominant order, the interaction energy depends exclusively on the distance r_{ij} separating the two lenses:

$$E_{\text{pair}} = \frac{m_i\, m_j\, g^2}{4\pi\sigma}\ln\left(\frac{r_{ij}}{R}\right) \qquad [43]$$

The expression of the interaction energy reveals that as far as the lenses motion is studied in the vicinity of the center of the soap film, the pair capillary attractive force only depends on the distance separating both lenses, and is directed along the line joining their centers,

$$\mathbf{F}_{\text{pair}} = -\boldsymbol{\nabla}(E_{\text{pair}}) = -\frac{g^2}{4\pi\sigma}\frac{m_i\, m_j}{r_{ij}}\,\mathbf{e_{ij}} \qquad [44]$$

where $\mathbf{e_{ij}}$ is the unit vector joining the lenses centers (see Fig. S4).

References

1. M Nicolson, The interaction between floating particles in Mathematical Proceedings of the Cambridge Philosophical Society. (Cambridge University Press), Vol. 45, pp. 288–295 (1949).
2. B Mercier, S Hamidouche, R Gautier, T Lacassagne, Educational Background Oriented Schlieren Based On A Matlab App And A Smartphone Camera in Proceedings of the International Symposium on the Application of Laser and Imaging Techniques to Fluid Mechanics. Vol. 20, (2022).

Movie S1. Two water lenses of 28 mg orbit around each other and merge on a horizontal circular soap film, viewed from above. The background speckle was intentionally made rough to facilitate naked-eye observation of the lenses and their merging process. Both lenses are injected onto the film using a hydrophobic slide on the right. For scale, the inner diameter of the frame holding the soap film is 10 cm. The frame rate has been adjusted so that the motion appears twice as slow as in real time.

Movie S2. Processed video of a single 28 mg water lens dropped onto one side of a horizontal soap film without any initial velocity. The inner diameter of the circular frame is 10 cm, and the video is in real time. Black regions indicate blurred zones in the original video, corresponding to areas where deformations of the soap film interface obscured the highly detailed speckle background.

Movie S3. Processed video of a single 28 mg water lens injected onto a horizontal soap film with an initial tangential velocity using a hydrophobic slide. The inner diameter of the circular frame is 10 cm, and the video is in real time. Black regions indicate blurred zones in the original video, corresponding to areas where deformations of the soap film interface obscured the highly detailed speckle background. We can see the lens orbiting around the center of the film and, every 7 seconds, reversing its direction of rotation. This is likely due to slight anisotropies in the experimental setup.

Movie S4. Real-time processed video of two 35 mg water lenses orbiting each other and merging. The first lens is deposited at the center of the film, while the second is injected on the side with a tangential velocity using a hydrophobic slide. The inner diameter of the circular frame is 10 cm. Black regions indicate blurred zones in the original video, corresponding to areas where deformations of the soap film interface obscured the highly detailed speckle background.

Movie S5. Real-time processed video of two water lenses of different masses orbiting each other and merging. The first lens (39 mg) is deposited at the center of the film, while the second (16 mg) is injected on the side with a tangential velocity using a hydrophobic slide. The inner diameter of the circular frame is 10 cm. Black regions indicate blurred zones in the original video, corresponding to areas where deformations of the soap film interface obscured the highly detailed speckle background. A significant difference in the lenses' dynamics is observed during the orbital phase, after which we see the lighter lens undergo extreme asymetrical distortions in the vicinity of the heavier one and during the merging process.

Movie S6. Processed video of two 28 mg water lenses orbiting each other and merging on a horizontal circular soap film, viewed from above. The video was processed from the original footage using the ComBOS software (2). For scale, the inner diameter of the frame surrounding the soap film is 10 cm. The frame rate has been adjusted so that the motion appears twice as slow as in real time. Lighter colors represent regions where the speckle background was distorted by the soap film and lens interfaces. The colors inside the lenses themselves are not relevant, as the background deformation is too strong to retain meaningful values.

Movie S7. Processed video of two 18 mg water lenses orbiting each other and merging on a horizontal circular soap film, viewed from above. The video was processed from the original footage using the ComBOS software (2). For scale, the inner diameter of the frame surrounding the soap film is 10 cm. The frame rate has been adjusted so that the motion appears twice as slow as in real time. Lighter colors represent regions where the speckle background was distorted by the soap film and lens interfaces. The colors inside the lenses themselves is not relevant, as the background deformation is too strong to retain meaningful values.
